# Age of Child, More than HPV Type, Is Associated with Clinical Course in Recurrent Respiratory Papillomatosis

**DOI:** 10.1371/journal.pone.0002263

**Published:** 2008-05-28

**Authors:** Farrel J. Buchinsky, Joseph Donfack, Craig S. Derkay, Sukgi S. Choi, Stephen F. Conley, Charles M. Myer, John E. McClay, Paolo Campisi, Brian J. Wiatrak, Steven E. Sobol, John M. Schweinfurth, Domingos H. Tsuji, Fen Z. Hu, Howard E. Rockette, Garth D. Ehrlich, J. Christopher Post

**Affiliations:** 1 Allegheny General Hospital, Allegheny-Singer Research Institute, Center for Genomic Sciences, Pittsburgh, Pennsylvania, United States of America; 2 Eastern Virginia Medical School, Norfolk, Virginia, United States of America; 3 Children's National Medical Center, Washington, DC, United States of America; 4 Children's Hospital of Wisconsin, Milwaukee, Wisconsin, United States of America; 5 Cincinnati Children's Hospital Medical Center, Cincinnati, Ohio, United States of America; 6 Children's Medical Center of Dallas, Dallas, Texas, United States of America; 7 The Hospital for Sick Children, Toronto, Ontario, Canada; 8 Departments of Human Genetics, and Microbiology and Immunology, Drexel University College of Medicine, Pittsburgh, Pennsylvania, United States of America; 9 Children's Hospital of Alabama, Birmingham, Alabama, United States of America; 10 Emory University School of Medicine, Atlanta, Georgia, United States of America; 11 University of Mississippi Medical Center, Jackson, Mississippi, United States of America; 12 Hospital das Clinicas of Sao Paulo University Medical School, Sao Paulo, Brazil; 13 Biostatistics Department, University of Pittsburgh, Pittsburgh, Pennsylvania, United States of America; Federal University of Sao Paulo, Brazil

## Abstract

**Background:**

RRP is a devastating disease in which papillomas in the airway cause hoarseness and breathing difficulty. The disease is caused by human papillomavirus (HPV) 6 or 11 and is very variable. Patients undergo multiple surgeries to maintain a patent airway and in order to communicate vocally. Several small studies have been published in which most have noted that HPV 11 is associated with a more aggressive course.

**Methodology/Principal Findings:**

Papilloma biopsies were taken from patients undergoing surgical treatment of RRP and were subjected to HPV typing. 118 patients with juvenile-onset RRP with at least 1 year of clinical data and infected with a single HPV type were analyzed. HPV 11 was encountered in 40% of the patients. By our definition, most of the patients in the sample (81%) had run an aggressive course. The odds of a patient with HPV 11 running an aggressive course were 3.9 times higher than that of patients with HPV 6 (Fisher's exact p = 0.017). However, clinical course was more closely associated with age of the patient (at diagnosis and at the time of the current surgery) than with HPV type. Patients with HPV 11 were diagnosed at a younger age (2.4y) than were those with HPV 6 (3.4y) (p = 0.014). Both by multiple linear regression and by multiple logistic regression HPV type was only weakly associated with metrics of disease course when simultaneously accounting for age.

**Conclusions/Significance Abstract:**

The course of RRP is variable and a quarter of the variability can be accounted for by the age of the patient. HPV 11 is more closely associated with a younger age at diagnosis than it is associated with an aggressive clinical course. These data suggest that there are factors other than HPV type and age of the patient that determine disease course.

## Introduction

It is now well established that human papilloma virus (HPV) causes recurrent respiratory papillomatosis (HPV) [1]. The most common types associated with the vast majority of RRP are HPV 6 and 11[2], [3]. While millions of children and adults are exposed, relatively few develop clinical evidence of the disease. The age of disease onset is highly variable and so is the clinical course [4]. The spectrum from indolent to aggressive is very wide. While the presence of HPV is necessary to develop RRP it is not sufficient by itself. We have previously postulated that genetically encoded host susceptibility plays a role in whether or not an individual develops RRP[5] and have started investigating *epidermodysplasia verruciformis 1* (*EVER1*) as a candidate gene [6]. The current study explores the other side of the host-pathogen interaction. Apart from host genetic factors, genetic variability intrinsic to viral genomes could also play a role in defining the clinical course of RRP. Previous studies have examined the relationship between HPV types and clinical disease behavior but the findings have not been consistently replicable. For example, the vast majority of previous studies have reported that human subjects infected with HPV 11 are more likely to develop an aggressive course of RRP compared to those infected with HPV 6 [7]–[13]. However, one study [14] revealed that HPV 6, not HPV 11, was more prevalent in children with aggressive RRP. Similar findings were reported with HPV 6 subtype C [15] (later shown to be HPV 11), and two studies reported no association between HPV type and clinical course [16]–[17].

As of April 2007, we have collected fresh, laryngeal biopsies from over 130 unique patients with RRP in collaboration with the RRP Task Force. To our knowledge, this collection of specimens represents the largest of its type in the world. The current study brings the largest dataset yet developed to bear on the potential relationship between clinical course and HPV type. Equally importantly, and often neglected, we explore how much of the variability of the disease can be attributed to the type of HPV while simultaneously accounting for other variables such as age.

## Results

Between January 2003 and April 2007 fresh papilloma biopsies were preserved in TRIzol® from 135 unique patients with RRP and were subjected to HPV typing. By using both allele specific PCR and RFLP typing on all cases we were able determine whether HPV 6 versus HPV 11 was present in the papilloma. Papillomas from 8 cases were positive for both HPV 6 and HPV 11 by both methodologies. Thus data was available on 127 unique patients who either had HPV 6 or HPV 11. Eight of the cases were excluded since we had less than one year of clinical course data (HPV 6 = 7, HPV 11 = 1, median age = 6.8y). Of the remaining 119 unique cases only one had adult-onset RRP and therefore was excluded. (Table 1)

**Table 1:** Data pertaining to 118 patients with RRP were subjected to further analysis.

Gender	male = 68 (58%), female = 50 (42%)
Race	White = 67 (57%), Black = 41 (35%), Asian, Other or Not Recorded = 10 (8%)
Hispanic ethnicity	Hispanic = 11 (9%), Non-Hispanic = 107 (91%)
Age at diagnosis	median = 3.0 years, range = (0.1, 13.1)
Duration of follow up (years)	median = 5.2 years, range = (0.9, 33)
Total number of surgeries	median = 19, range = (2, 402)
Max number of surgeries in a 12 month period	median = 4, range = (1, 52)
Interval since previous surgery	median = 113 days, range = (10 days, 3 years)
Distal Involvement	involved = 30 (25%), uninvolved = 88 (75%)
Tracheostomy	currently or previously = 12 (10%), never = 106 (90%)
Aggressiveness of course	aggressive = 95 (81%), indolent = 23 (19%)
HPV type	HPV 6 = 71 (60%), HPV 11 = 47 (40%)

Subjects who had both HPV 6 and 11 present and subjects for whom there was less than 1 year of clinical data were excluded. The descriptive statistics are stated.

Initial analysis was directed at determining whether or not HPV type and clinical course were associated or independent of each other. There was indeed an association between HPV 11 and a more aggressive course (Table 2). “Aggressiveness” was a label based on a composite of four separate criteria. The labeling, also called coding, was decided upon before subjects were enrolled in the study. The four individual criteria making up the composite label are defined in the methods section: total number of surgeries, frequency of surgery, distal involvement, tracheostomy status. Not only did we analyze the composite clinical course as a function of the HPV type but we also sought an association between HPV type and each separate criteria (Table 3). The odds ratio for total number of procedures was 2.6 (95% CI 1.0 to 7.5) for subjects with HPV 11 as opposed to those with HPV 6, for maximum annual frequency it was 2.0 (95% CI 0.86 to 4.9), for tracheostomy status it was 3.4 (95% CI 0.84 to 16) and for distal involvement the odds ratio was 3.7 (95% CI 1.4 to 9.8) with 40% of the HPV 11 cases having had distal involvement as opposed to 15% of HPV 6 cases. Distal involvement frequently portends a severe clinical course but none more so than that subgroup which has pulmonary involvement. In the sample of 118 cases, 6 had pulmonary involvement. Three of the 6 cases were infected with HPV 6 and the other 3 had HPV 11. We did not specifically collect data regarding malignant transformation but no such information was volunteered on the data collection forms for the 118 patients.

**Table 2:** Contingency table of HPV type versus nature of clinical course.

	indolent	Aggressive	odds ratio (p-value)
HPV 6	19	52 (73% of all HPV 6 cases)	
HPV 11	4	43 (91% of all HPV 11 cases)	3.9 (0.02)

Fisher's exact test yielded a p-value of 0.017 with odds ratio of 3.9 (95% CI 1.2 to 17) and a risk difference of 28%. A clinical course was defined as “aggressive” if any of the following criteria were met: total procedures ≥10, procedure frequency ever ≥4 per year, ever had distal involvement, ever had a tracheostomy tube.

**Table 3:** Contingency table of HPV type versus four individual criteria that taken together constituted the composite “aggressiveness” label.

Surgery Count	total procedures <10	total procedures ≥10 (row percentage)	odds ratio (p-value by Fisher Test)
HPV 6	25	46 (64% of all HPV 6 cases)	
HPV 11	8	39 (83% of all HPV 11 cases)	2.6 (0.04)
**Max Surgery Frequency**	procedure frequency never ≥4 per year	procedure frequency ever ≥4 per year	
HPV 6	31	40 (56%)	
HPV 11	13	34 (72%)	2.0 (0.08)
**Distal Involvement**	distal involvement: never	has had distal involvement	
HPV 6	60	11 (15%)	
HPV 11	28	19 (40%)	3.7 (0.004)
**Tracheostomy**	tracheostomy: never	has had tracheostomy	
HPV 6	67	4 (6%)	
HPV 11	39	8 (17%)	3.4 (0.06)

Rather than simply looking at the data dichotomously (aggressive vs. indolent) the total number of surgeries were analyzed as a count variable. Total number of surgeries thus far in the clinical course was log transformed since the untransformed variables were highly skewed. The geometric mean number of surgeries in the cases with HPV 6 was 16 and in the cases with HPV 11 it was 30 (t-test p-value = 0.005) suggesting that subjects infected with HPV 11 tend to follow an aggressive course of the disease.

The above data not only represents yet another set supporting the association between HPV 11 and aggressive course but it is also the largest data set to ever be analyzed. At this point one may conclude the case finally settled and resolve to use HPV typing as a clinical prognosticator. Yet that would be premature since other variables, and not just HPV type may be associated with clinical course.

**Figure 1:**
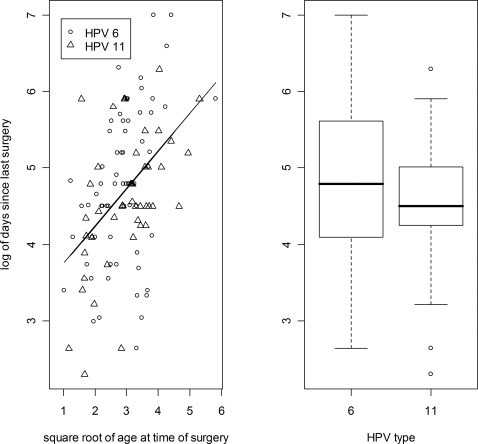
Papilloma biopsies were taken at the time of enrollment and subjected to HPV typing. For those cases where the enrollment surgery was not their first surgery, the interval in days since the last surgery was recorded. Variables were transformed so that they would comply with a Gaussian distribution. The natural log of the number of days since the last surgery are depicted on the y axis. The left panel depicts a linear regression against the square root of the age at the time of enrollment. The model predicts that a 4 year old last had surgery 69 days (2.3 months) ago and a 9 year old last had surgery 113 days (3.7 months) but only 20% of the variability is accounted for by this model. The right panel depicts a boxplot of the same data but clustered by HPV type. The geometric mean of the number of days since the last surgery was 119 days (3.9 months) for those with HPV 6 and a similar 97 days (3.2 months) for those with HPV 11. The t-test p-value was 0.25.

Gender and Race are categorical variables just as HPV type is. Yet there was no association between them and disease course. Age can be cut into a dichotomous variable but with no obvious cut point. The variable fitted a Gaussian distribution best when it was transformed by taking the square root. Thus the mean age at the time of diagnosis of the children who had experienced an aggressive course was 2.7 years, but was 4.3 years for those with an indolent course (t-test p-value = 0.01). For the 6 cases with pulmonary involvement, the mean age of diagnosis(based upon square root transformed data) was 1.3 years for those affected and 3.1 years for those with no recorded pulmonary involvement (t-test p-value = 0.002). The range of age of diagnosis for those with pulmonary involvement was 0.5 to 2.6 years. In other words, all 6 cases fell below the 42nd percentile for the sample as a whole. While age of diagnosis has been associated with aggressiveness as measured by the frequency of surgery, Reeves et al. [18] demonstrated quite clearly that surgical frequency was more a function of the age of the child at the time that the frequency is being measured, than the age of the child at the time of diagnosis. Our data collection method differed from that of Reeves et al. and therefore the current analysis investigated the frequency by looking at the number of days since the surgery prior to the surgery at which the patient's papilloma biopsy was taken. As Reeves et al. found, there was indeed a correlation between the age at enrollment and the interval since the last surgery. Linear regression revealed that 20% of the variability in the interval since the last surgery was a function of the age at enrollment (p = 4*10^−7^). On the other hand, HPV type was not related to the interval since the last surgery (t-test p-value = 0.25). (Figure 1)

It is quite conceivable that the duration between any two surgeries may be a function of more than just the current aggressiveness of the disease. Some surgeons schedule subsequent surgery as symptoms dictate, whereas other surgeons set a pre-determined duration based upon the agressiveness of the clinical course over the preceding year. Others have adopted a hybrid of the two methods. The variation in practice style was controlled for by coding the surgeon's response for each patient as 1 for “as required (prn)”, 2 for “hybrid” and 3 for “predetermined”. Adding the surgeon's style to the linear model accounted for an additional 7% of the variability (p-value = 0.010). Age at diagnosis was added to the model and further accounted for some of the variability in a statistically significant manner (p-value = 0.014) albeit that it only accounted for 6% of the variability. So while age at diagnosis was correlated with the duration since the preceding surgery, this correlation was very weak when contrasted against that of age at the time of enrollment in the study. Together, age at enrollment+age at diagnosis+surgeon's style of determining surgical interval accounted for 31% of the variability in the time since the preceding surgery. Adding HPV type accounted for none of the residual variability. Thus in the multiple linear regression equation where y = log (days between last and second last surgery), coefficients were calculated for 4 variables simultaneously; in other words it was not a step-wise hierarchical model where a subsequent term is allowed to explain the residual left over after a preceding term has already been entered into the model. The p-values for each of the estimated coefficients were as follows: HPV type = 0.95, square root of at age of enrollment = 6×10^−5^, square root of age at diagnosis = 0.015 and surgeon's style = 0.013. Fourteen observations were excluded either because the surgeon's style was missing or the patient had been enrolled at the time of the first surgical intervention and thus there was no duration between the last and the second last surgery. Similarly, mother's highest educational qualification and gross household income accounted for none of the variability. Even the anatomical component of the Derkay/Coltrera score at the time of enrollment did not account for the variability in the time since the last surgery given that the age variables and the surgeon's style had already been accounted for.

Since the duration between surgery at the time of enrollment in the study and the preceding surgery utilizes information from only a small interval of the subject's clinical course it may be highly variable. Thus, a similar analysis was conducted using the annual frequency of surgeries around the time of the enrollment. The sampling period was a median of 1.8 years (range 0.9 to 2). As above, the age was transformed to the square root and the frequency of procedures in the time period surrounding enrollment was log transformed. Results were similar to that obtained above; a simple linear regression model declared that square root of the age at the time of enrollment accounted for 20% of the variability in the natural log of the annual frequency of surgeries around the time of enrollment (p = 4.6*10^−7^). Together, age at enrollment+age at diagnosis+surgeon's style of determining surgical interval accounted for 37% of the variability in the time since the preceding surgery. However, unlike for the variability in duration since preceding surgery, annual frequency variability was very highly significantly correlated with both age variables: age at time of measuring frequency p-value = 0.00023; age at time of diagnosis p-value = 0.00020. Adding HPV type once again accounted for none of the residual variability.

We have seen above that HPV 11 is associated with a more aggressive clinical course as is young age of the patient, with clinical course being far more correlated with age than with HPV type. In fact HPV type is correlated with age of the patient and this is why one sees an association between HPV type and clinical course, if not simultaneously controlling for the age of the patient. This is most easily seen if one performs logistic regression with clinical course measured as a binomial variable as defined above. Children with HPV 11-induced disease are diagnosed at a younger age than children with HPV 6; the average age of diagnosis amongst those with HPV 6 was 3.4 years and the average age of diagnosis for those with HPV 11 was 2.4 years (t test p-value = 0.014). Logistic regression in which HPV-type was stipulated as the sole independent variable accounted for 5.6% of the deviance (p-value = 0.02) and when age of diagnosis (transformed to square root) was the sole independent variable, it accounted for 7.4% of the deviance (p-value = 0.005). When the two variables were handled simultaneously in a multiple logistic regression then 12% of the deviance was accounted for with the individual p-values being 0.057 for HPV Type and 0.017 for age of diagnosis.

## Discussion

RRP is a devastating disease-sometimes because of the urgency of symptoms at presentation (such as respiratory distress), but far more commonly because of the chronicity of the disease and the multitude of surgeries undertaken. Amongst the many patients with RRP there is considerable variability in the clinical course. For many years investigators have explored the relationship between the clinical course, and whether the patient was infected by HPV type 6 or type 11, but many studies suffered from small sample size. Now, thanks to a large multi-center collaboration that was founded to explore the underlying genetic susceptibility to RRP, we have access to the largest dataset ever assembled. With confidence one can note that HPV 11 is associated with a more aggressive disease but that the clinical course is more closely associated with the age of the child which in turn is associated with HPV type.

Younger children tend to have a more aggressive clinical course and so do children with HPV 11. However, HPV type is associated with both the age of the child and with the outcome, namely an aggressive clinical course. Once the age of the child is controlled for, then the relationship between HPV type and clinical course becomes one of borderline significance. We can speculate upon the association between the age of the child and frequency of surgery. Investigators have believed that the relationship is due to the relatively small airway of younger children demanding more frequent surgery to keep it clear. In our study we did not measure the diameter of the airway following papilloma removal. The closest approximation to such a value can be garnered from an article in which appropriate endotracheal tube diameter was discussed for each age [19]. Such measurements probably reflect the diameter of the subglottis. In our data the estimated size of the airway did not account for the variability in annual frequency once age was already in the model. Others have speculated that the developing immune system may permit older children to have slower papilloma growth. Although HPV 11 does not appear to be more virulent with respect to the clinical course, the fact that it does affect those who are diagnosed at a younger age may be a sign of virulence in and of itself. Since the exposure for all cases was at the time of birth, the data herein demonstrates that the incubation period for HPV 11 (2.4y) is significantly shorter than for HPV 6 (3.4y).

Previous studies [18] have also shown that the age of the patient is associated with the clinical course. The data collected by the RRP Task Force in collaboration with the Centers for Disease Control and Prevention was extensive. Given that RRP is a rare disease it was a feat to have collected the data of 603 unique cases. A thorough analysis of the data demonstrated the association with age quite convincingly. The study was based on a medical record review and since papilloma specimens were not obtained, HPV type was unknown. Similarly, Silverberg et al. [4] also noted more frequent surgery in younger children and noted that most children (two thirds) underwent less frequent surgery over time. Only 14% of the 42 children underwent more frequent surgery in later years than that following diagnosis. Neither Reeves et al. nor Silverberg et al. could comment on HPV type, but other studies have shown that younger children seem to be more frequently infected by HPV 11 [10]. Multiple regression, be it linear or logistic, provides a way to explore the three-way relationship of clinical course, age and HPV type.

A possible criticism of our findings is that we have analyzed a biased sample of aggressive RRP cases based upon our acquisition process. The data described herein was acquired for the principal purpose of determining the underlying genetic susceptibility to RRP. The design is cross-sectional and acquires probands at any stage of their disease. Our enrollment mechanism was unlikely to reach cases diagnosed a long time ago who subsequently had an indolent course and went into remission before our study was initiated. On the contrary those who were diagnosed at a similar time but have endured an aggressive and protracted course would have been reached by our acquisition mechanism. Furthermore, we excluded 8 cases from analysis since the duration of their follow up had been shorter than a year. These factors combined, bias our sample to a generally more aggressive clinical course than the population of all incident cases tracked over their lifetime. Another weakness of this cross-sectional study is that data was not collected from each and every surgical intervention. Rather, physical findings were recorded only at the time of enrollment and summary data pertaining to history were recorded at enrollment and 1 year later. An ideal metric of papilloma burden would have been a documentation of the full Derkay/Coltrera Staging Score for each individual from the time of diagnosis to the time of remission. The area under such a curve would be less subject to stochastic phenomena in each individual's course.

Less than half of RRP surgeons send papillomas for HPV typing [20]. It is common for laboratories to only differentiate “high risk” from “low risk” as if risk was being determined for cervical cancer. In other words, routine laboratory testing will differentiate HPV 16 from HPV 6/11; only highly specialized laboratories or research laboratories would differentiate HPV 6 from HPV 11. The vast majority of cases in this study were managed by surgeons who were unaware of the HPV type and thus those deciding when to operate were blinded with respect to HPV type.

Given the variability of the clinical course it would be useful to be able to predict when a particular child would require subsequent surgery. The current study suggests that HPV type would be of no or little utility in making this determination. Age of the patient at the time and age of the patient at diagnosis would be more accurate predictors but even with these factors taken into effect we could only account for about one third of the variability.

Malignant transformation of RRP is a devastating but rare complication that has been investigated by others. Malignancy was not specifically addressed by the incumbent study. Nevertheless, it is important for the reader to be aware that other groups have investigated the features of malignant transformation and have associated them with pulmonary involvement and with HPV 11 exclusively (that is no isolates of HPV 6 from malignant RRP biopsies) [12], [21].

The cohort described herein had a similar rate of co-infection compared to that which has already appeared in the literature. In the initial cohort of 135 cases (before exclusions for insufficient clinical course data) for whom we had fresh papilloma specimens, only 8 (6%) were deemed to harbor both HPV 6 and 11. Following exclusion of cases with insufficient clinical course data there were only 5 cases with co-infection; statistical analysis of such a small group would be of questionable meaningfulness. Furthermore, one would not know if both HPV types were leading to the phenotype since it is conceivable that one of the types had caused the disease and the other was merely present much in the same way that HPV 6 and 11 have been found in the mucosa of people without RRP. Our method of typing used two methods on each specimen (allele-specific PCR and RFLP). Co-infection rates between papers could be difficult to interpret since methodologies vary from study to study. (Table 4)

**Table 4:** Listing of literature in which HPV typing was reported.

Study	HPV 6	HPV 11	coinfection
Rabah 2001[10]	29 (48%)	32 (52%)	0 (0%)
Wiatrak 2004 [11]	31 (53%)	23 (40%)	4 (7%)
Gabbott 1997 [16]	19 (43%)	24 (55%)	1 (2%)
Pou 1995 [8]	21 (88%)	2 (8%)	0 (0%)
Draganov 2006 [13]	6 (26%)	14 (61%)	3 (13%)
Padayachee 1993[14]	5 (25%)	15 (75%)	0 (0%)
Rimell 1997 [9]	9 (47%)	6 (36%)	4 (21%)
Szeps 2005 [28]	12 (66%)	6 (33%)	0 (0%)
Maloney 2006[29]	4 (27%)	4 (27%)	7 (47%)
Pou 2004 [30]	6 (46%)	7 (54%)	0 (0%)
**Total**	**142 (48%)**	**133 (45%)**	**19 (6%)**

Studies where co-infection rate could not be ascertained are not included below. Articles are listed in descending order of sample size. The row entitled “Total” assumes that there was no overlap in the cohorts described. Some studies noted other HPV types (such as HPV 16 in someone who had undergone malignant transformation) and are not reported in this table.

The RRP Task Force and the Center for Genomic Sciences, Allegheny-Singer Research Institute in Pittsburgh, PA are currently collaborating to determine the underlying genetic susceptibility in RRP. A secondary objective is to determine what the genetic factors are that account for the variability in clinical course. One of the reasons for obtaining a papilloma specimen at the time of enrollment was to determine whether the patient was infected with HPV 6 or 11. We believed that HPV type would be an important factor to control for in trying to observe an association between aggressiveness and underlying genotype. Given the data contained in this report it appears as if HPV type may not be a significant variable, or if it is, then only weakly so. While it is not known what factor(s) account for the remaining two thirds of the variability in clinical course, a reasonable hypothesis would be that host genetic factors account for the remainder of the clinical variability. A genome wide search for those factors is underway.

## Methods

### Sample composition

The RRP patients studied here were identified through an ongoing collaborative study on the genetics of RRP with the coordinating site located at Allegheny-Singer Research Institute, Allegheny General Hospital, Pittsburgh, PA [5]. As of April 2007, 19 institutions have approved the study and thus far 15 have contributed laryngeal specimens. People diagnosed with RRP and managed by an otolaryngologist were invited to participate in the study. At first the study was limited to pediatric patients and later was opened to adult patients. The genetic susceptibility study has two acquisition routes: satellite site design and patient-support group design. In the satellite site design, cases were enrolled by their attending otolaryngologist and laryngeal papilloma, blood and clinical data were submitted. In the patient-support group design, interested potential subjects contacted the principal investigator. Arrangements were made for the subjects to submit their clinical data and a Scope® mouthwash specimen; no papilloma specimens were obtained. Clearly, those subjects enrolled through the patient-support group design could not be included in the subset analyzed herein. The protocol was originally approved by the institutional review board (IRB) of the Allegheny-Singer Research Institute, Pittsburgh, PA. In an iterative process the protocol was subsequently submitted to 20 separate IRBs over a 5 year period and approval was obtained in the following order: Children's Hospital of the King's Daughters, Norfolk, VA; Children's Hospital of Wisconsin, Milwaukee, WI; Children's National Medical Center, Washington, DC; Cincinnati Children's Hospital Medical Center, Cincinnati, OH, Johns Hopkins Children's Center, Baltimore, MD; Children's Medical Center of Dallas, Dallas, TX; Children's Hospital of Philadelphia, Philadelphia, PA; SUNY Upstate Medical Center, Syracuse, NY; The Hospital for Sick Children, Toronto, ON, Canada; Children's Hospital of Alabama, Birmingham, AL; University of California-San Francisco, San Francisco, CA; Children's Hospital of Pittsburgh, Pittsburgh, PA; Le Bonheur Children's Medical Center, Memphis, TN; Children's Healthcare of Atlanta at Egleston, Atlanta, GA; Bastian Voice Institute, Downers Grove, IL; British Columbia's Children's Hospital, Vancouver, BC; University of Mississippi Medical Center, Jackson, MS; Hospital das Clínicas da Faculdade de Medicina da Universidade de Sao Paulo, Sao Paulo, Brazil; UPMC Presbyterian Hospital, Pittsburgh, PA; Yale-New Haven Children's Hospital, New Haven, CT. No IRB was prepared to engage in a cooperative review agreement as allowed for in section 114 (45CFR46) the code of federal regulations [22]. All human subjects or a parent (or legal guardian) provided written informed consent. Written assent was obtained from children in accordance with specific institutional practice.

### Clinical Course

Information about the clinical course of each case was supplied by their otolaryngologist at the time of enrollment and one year later updated clinical information was supplied. Metrics of clinical course included total number of airway procedures (count), frequency of procedures (count per year), ever had involvement in the trachea or distally (binomial), ever had a tracheostomy (binomial). A clinical course was defined as “aggressive” if any of the following criteria were met: total procedures ≥10, procedure frequency ever ≥4 per year, ever had distal involvement, ever had a tracheostomy tube. If none of these criteria were met the patient was considered to be experiencing an indolent course. Numeric cuttoffs can appear arbitrary but the metrics used in this study are based on precedence set by other investigator groups and importantly, were decided upon a priori. Doyle et al, working in New Orleans and Derkay, reporting a national survey of otolaryngologists, used identical or similar cut offs for classifying aggressive versus benign [23], [24]. Their classification metrics have been referred to by others and have been used by others [25], [26].

For each patient the surgeon was asked about the manner in which he or she decided to perform the next surgical intervention. The exact wording on the forms was, “Some surgeons operate on patients according to pre-determined intervals. Others only return to the operating room on a prn (as required) basis. Some adopt a hybrid approach whereby only the next interval is pre-determined in response to what happened over the last one or two intervals. What approach are you closest to with respect to this patient over the past year? “The surgeon's response for each patient was coded as 1 for “as required (prn)”, 2 for “hybrid” and 3 for “pre-determined”.

### Specimen collection and DNA extraction

During surgery to control the patient’s disease, two 1mm by 1mm papilloma biopsies were collected by the patient's attending physician and preserved in 700 µl of TRIzol® solution contained in a 1.5 ml microcentrifuge tube. The biopsy specimens were shipped to Allegheny-Singer Research Institute, Pittsburgh, PA, the specimen and data coordinating site. DNA was extracted from laryngeal biopsies according to the manufacturer's guidelines (Invitrogen, Carlsbad, California). To assess the quantity and quality of the isolated DNA, samples were tested by spectrophotometry using a ND-1000 spectrometer (NanoDrop) and by PCR using the GH20 (5′-GAAGAGCCAAGGACAGGTAC-3′) and PC04 (5′-CAACTTCATCCACGTTCACC-3′) primers which span a 268 bp segment of the β-globin gene [27]. During the early phase of establishing the specimen collection, several samples failed to yield β-globin amplicons. Thus the specimens that failed PCR were subjected to whole genome amplification (WGA) using the GenomiPhi DNA Amplification Kit from Amersham Biociences (Piscataway, NJ) following the manufacturer's instructions. After the extraction of the first 45 laryngeal specimens, we decided to systematically carry out WGA on all extracted DNA. The products of the WGA reactions were tested by seeking the presence of the 268 bp PCR fragment of the β-globin gene using GH20 and PC04 primers.

### HPV Typing

DNA was analyzed for the presence of HPV sequences by both Type-Specific PCR primers (TS-PCR) and Restriction Fragment Polymorphism (RLFP) on all laryngeal specimens. These two HPV typing techniques were systematically used in our specimens to ensure reproducibility and accuracy of genenotyping results. In fact, like any other PCR-based genotyping, TS-PCR is very sensitive and can produce either false positives or false negatives, which is a potential explanation for the very high frequency of dual infection reported in some RRP studies. RLFP typing using a different set of PCR primers followed by specific endonuclease digestion was used to replicate TS-PCR results and confirm HPV genotype.

### Type-Specific PCR

**Figure 2:**
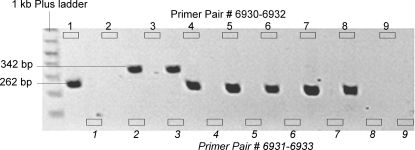
Agarose gel electrophoresis of HPV 6 and 11 specific PCR fragments. The first 2 lanes after the ladder (both labeled “1”) represent the wells for HPV 6 positive control clone for the TS-PCR using either primer pair 6930–6932 (designed to be specific for HPV 6) or 6931–6933 (designed to be specific for HPV 11). The same pattern applies to the remaining lane couplets. The couplet labeled “2” represents HPV 11 positive control clone. Couplets 3–8 are RRP specimens and couplet 9 is the PCR negative control. In this gel we see that that laryngeal DNA used in couplet 3 contained only HPV 11 and the remainder contained only HPV 6.

For each HPV type, two Type-Specific (TS) primers were designed in the E6 (HPV 6) and L2 (HPV 11) region. Prior to selecting the primers, HPV 6 and HPV 11 DNA sequences were downloaded from the public database at Los Alamos National Laboratory (http://hpv-web.lanl.gov/). These sequences were aligned using BLAST 2 SEQUENCES (http://www.ncbi.nlm.nih.gov/blast/bl2seq/wblast2.cgi) to reveal highly conserved and discordant regions between the two types. Only areas of high DNA sequence discordance were used to design primers using Oligo, a primer analysis software V.6.65 (Molecular Biology Insights, Inc., Cascade, CO). The sequences of primers that best met Oligo PCR primers design criteria and empirical annealing temperature were as follow; HPV 6-specific primers: forward primer (6930) 5′-ACCTAAAGGTCCTGTTTCGAGG-3′, reverse primer (6932) 5′-CAGCGACCCTTCCACGTAC-3′ and HPV 11: forward primer(6931) 5′-ATATACCCTTGGGAAGCTCTCC-3′, reverse primer(6933) 5′-TTGTTGGGGCAGATATAAGTATG-3′. These primers were blasted against public genomic databases to ensure they matched to HPV 6 and 11 with high probability scores. To empirically determine the optimal annealing temperature for each primer set, a gradient PCR was then carried out separately using each TS primer and HPV 6 and 11 cloned DNA as template DNA (generously provided by Drs. P. Reidy and W. Lancaster from Department of Otolaryngology, Wayne State University, Detroit, MI). Annealing temperatures used were 67°C and 68°C for both primers and AmpliTaq Gold® DNA Polymerase from Applied Biosystems (Foster City, CA). HPV positive and negative control reactions were included in all HPV TS-genotyping experiments. The HPV-positive controls consisted of HPV 6 and HPV 11 cloned genomic DNA. PCR products were subjected to electrophoresis on 1% agarose gels, and visualized by ethidium bromide staining (Figure 2).

### Restriction Fragment Length Polymorphism (RLFP)

**Figure 3:**
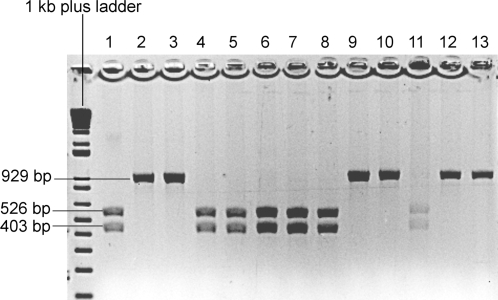
HPV 6 and 11 genotyping by restriction fragment length polymorphism. *BstN* I enzyme is a single cutter for the 929 bp HPV6 and 11 consensus PCR fragment. HPV6 possesses the enzyme recognition site thus the digestion yields two fragments of size 526 and 403 bp while HPV11 does not. Lanes 1 and 2 are HPV 6 and 11 positive controls. Lanes 3–13 are RRP patients.

HPV6 and 11 consensus E7 and E1 primers Forward primer: 5′-TGACCTGTTGCTGTGGATGT-3′ and reverse primer 5′-GTGCATATAAACTTAATGGCTCAA-3′ were used to amplify a 929 bp DNA segment. PCR products were digested at 37°C overnight using enzyme *BstN* I (New England Biolabs, Ipswich, MA) following the manufacturer's protocol. The products of the digestion were run on 1% agarose gel and visualized by ethidium bromide staining (Figure 3).

### Statistical Analysis

Prior to any subject enrollment the primary metric of clinical course was defined as the composite label “aggressive” or “indolent” as stated above under “clinical course”. While clinical course was considered a dependent outcome measure, the following variables were considered to be independent potential predictor variables: HPV type, age at diagnosis, age at enrollment, gender, race, educational status of mother, gross household income, surgeon's style. The dataset was initially inspected by conducting a descriptive statistical summary of all the variables in isolation (R statistical software (http://www.r-project.org/)). Boxplots and histograms of each variable were inspected. Several variables did not follow a Gaussian distribution and thus were either transformed by taking the natural logarithm or by taking the square root of the value. Age of diagnosis, age at enrollment, total number of procedures, frequency of procedures per year, time from last operation to time of current operation and gross household income were transformed. Initial analysis consisted of performing a Fisher exact test of independence for the two binomial variables: HPV type (6 vs 11) and clinical course (aggressive vs indolent). Secondarily, multiple logistic regression was then used to analyze two independent variables that had been repeatedly described in the literature; HPV type and the age of the subject were simultaneously related to the dependent (outcome) variable of clinical course (aggressive vs. indolent). Finally, in a post-hoc manner, the 8 independent variables described above were explored in models in which total number of surgeries, frequency of surgery in a one year period and time between last and second last surgery were modeled as continuous variables. The purpose of the models was to determine the fraction of the variability in the outcome that could be accounted for by the known independent predictor variables. Specifically, we sought the contribution of the HPV type in accounting for clinical course variability in the context of other known variables. No adjustments have been made for multiple testing.
